# Diagnosis and application of rice diseases based on deep learning

**DOI:** 10.7717/peerj-cs.1384

**Published:** 2023-06-13

**Authors:** Ke Li, Xiao Li, Bingkai Liu, Chengxin Ge, Youhua Zhang, Li Chen

**Affiliations:** 1 School of Information & Computer, Anhui Agricultural University, Hefei, Anhui, China; 2 Anhui Agricultural University, Anhui Provincial Engineering Laboratory for Beidou Precision Agriculture Information, Hefei, Anhui, China; 3 Anhui University, Information Materials and Intelligent Sensing Laboratory of Anhui Province, Hefei, Anhui, China; 4 School of Plant Protection, Anhui Agricultural University, Hefei, Anhui, China

**Keywords:** Rice disease, Deep learning, YOLOV5s, Neural network

## Abstract

**Background:**

Rice disease can significantly reduce yields, so monitoring and identifying the diseases during the growing season is crucial. Some current studies are based on images with simple backgrounds, while realistic scene settings are full of background noise, making this task challenging. Traditional artificial prevention and control methods not only have heavy workload, low efficiency, but are also haphazard, unable to achieve real-time monitoring, which seriously limits the development of modern agriculture. Therefore, using target detection algorithm to identify rice diseases is an important research direction in the agricultural field.

**Methods:**

In this article a total of 7,220 pictures of rice diseases taken in Jinzhai County, Lu’an City, Anhui Province were chosen as the research object, including rice leaf blast, bacterial blight and flax leaf spot. We propose a rice disease identification method based on the improved YOLOV5s, which reduces the computation of the backbone network, reduces the weight file of the model to 3.2MB, which is about 1/4 of the original model, and accelerates the prediction speed by three times.

**Results:**

Compared with other mainstream methods, our method achieves better performance with low computational cost. It solves the problem of slow recognition speed due to the large weight file and calculation amount of model when the model is deployed in mobile terminal.

## Introduction

Rice is an important agricultural crop. However, rice diseases will significantly reduce its yield and quality, posing a major threat to food supply around the world (Sethy et al., 2020). Therefore, disease control is extremely important for rice production. The key of disease control is to diagnose it correctly and quickly, so that pesticide control measures can be taken in time. At present, the most widely used method for diagnosing rice crop diseases is manual judgment based on disease appearance. As not enough people have the skills to perform such tasks in time, a more effective and convenient method for diagnosing rice diseases is needed.

Disease diagnosis through visual observation of symptoms can be complicated due to the similarity of symptoms between various diseases. This complex nature can even confuse experienced personnel to incorrectly identify diseases, which can lead to further problems. These methods can be divided into two categories. The first type of method develops new features and image sets for classification that are not recognizable to the naked eye. For example, Feng et al. (2020) fused three different types of spectral datasets with visible/near-infrared hyperspectral imaging (HSI), mid-infrared spectroscopy (MIR) and laser-induced breakdown spectroscopy (LIBS) to detect three different rice diseases, namely leaf blight, rice blight, and rice sheath disease. The classification models used support vector machine (SVM), logistic regression (LR) and convolutional neural network (CNN) models. The HSI-based model achieved the best results with an accuracy of more than 93%. Lu et al. (2022) proposed an automatic rice disease classification and recognition framework. The improved DBN and SPSO-SVM can simultaneously analyze three features, including color, texture and shape, in order to identify disease types from regions of interest obtained from preprocessed disease images. But how to find the best weight and deviation parameter is the problem of this method. Based on the improved mean shift image segmentation algorithm, Liu et al. (2014) can segment 15 kinds of rice disease spots from the original image, and use SVM model to accurately classify the disease. However, due to the small amount of data and the inconspicuous characteristics of striped leaf blight, the identification of individual diseases is not accurate enough. Joshi & Jadhav (2016) developed an algorithm to extract the shape and color features of the diseased part of leaves and used MDC and KNN to classify the diseases. This method has advantages in terms of time complexity and accuracy but its recognition ability is relatively targeted and universal. It only achieves good results for the four diseases in this article. The advantages of machine learning are that the algorithm is simple and easy to understand, quick to implement, friendly to small-scale data, suitable for multi-classification tasks, easy to explain, and few parameters to be adjusted. However, the recognition speed is slightly slow, and the accuracy of recognition needs to be improved.

The second type of methods improved existing CNN models to achieve a better performance. Jiang et al. (2021) improved VGG16 model with the idea of multi-task learning and then used the pre-training model on ImageNet to transfer learning and alternate learning. The training time of the model is longer than that of the single model, but the accuracy is significantly improved. Zhou et al. (2019) proposed a fast rice disease detection method based on the fusion of FCM-KM and Faster R-CNN, which solved the problem of the original K-Means algorithm felling into local optimum, improved the recognition accuracy, and reduced the recognition time. However, this method is not suitable for monitoring large-scale rice cultivation. Rahman et al. (2020) proposed a two-stage small CNN architecture, which fine-tuned the pre-trained ImageNet weights and provided the best results for all five methods to detect and identify rice pests and diseases. However, the network model is large in scale and needs sufficient hardware resources to realize it. Chen et al. (2021) adopted Mobilenet-V2 pre-trained on ImageNet as the backbone network, added attention mechanism and optimized loss function, and carried out two ways of transfer learning. It enhances the ability to learn the characteristics of minor diseases, so that the diseases can be found in time at the initial stage. However, some different rice diseases occurring in the same plant affect the classification results. On the basis of ResNet and Shufflenet-V2, Kalpana & Karthiba (2016) proposed PD2 SE-NET to assist CNN-based automatic estimation of rice disease severity, but this model has certain deficiencies in fine quantitative evaluation and accurate evaluation. Lu et al. (2017) improved the traditional CNN to enhance the learning ability of the model and could effectively classify 10 common rice diseases. This method has better recognition ability and faster convergence rate than other models. However, there are too many parameters in CNNs, so finding the optimal parameters is a very challenging problem, and the time cost is higher than that of other models. Zhang & Zhang (2019) proposed a method of rice blast feature extraction and disease classification based on deep convolutional neural network (CNN), established different rice disease recognition systems, and realized automatic rice disease diagnosis. However, the common data enhancement methods are not effective, and the robustness of the system is poor, so it is easily affected by environmental noise.

In conclusion, the advantages of deep learning include good portability, high recognition accuracy, fast recognition speed and strong learning ability. However, the hardware requirements of deep learning are high, and the training model has many parameters, long period and large amount of data. Therefore, it is necessary to develop a lightweight network suitable for small-scale data sets for application in rice disease diagnosis. Existing research on the use of deep learning for rice diseases dealt with a limited number of rice diseases. The more effective target detection network in the current YOLO (You Only Look Once) series is YOLOV5. It is a generation of network that is based on the improvements and innovations of YOLOv4, and is far more flexible and faster than YOLOv4, with extremely strong advantages in the rapid deployment of the model. The authors who proposed the YOLOV5 detection network provided four network models: YOLOV5x, YOLOV5l, YOLOV5m, and YOLOV5s. Among them, the YOLOV5s network has the smallest feature map width and convolution depth in the entire YOLOV5 series, and the other three different models are continuously deepening the convolution and widening the feature maps based on YOLOV5s. The comparison of YOLOV5 series models can be seen in Table 1. The overall performance of YOLOV5s is more balanced, with a higher mAP value, smaller model memory after training, faster model loading, and faster target detection speed. Therefore, YOLOV5s of the YOLOV5 series was chosen as the basic model for the research on rice disease detection tasks.

**Table 1:** The comparison of YOLOV5 series models.

Model	Size (pixels)	mAP (%)	Speed CPU(ms)	Speed GPU(ms)	Params (M)	FLOPs (B)
YOLOV5s	640	56.8	98	0.9	7.2	16.5
YOLOV5m	640	64.1	224	1.7	21.2	49.0
YOLOV5l	640	67.3	430	2.7	46.5	109.1
YOLOV5x	640	68.9	766	4.8	86.7	205.7

YOLO is a first-order target detection model. Compared to second-order structures such as Faster RCNN, the YOLO network uses a first-order structure to perform target detection by directly predicting the class and position of the target, which has the advantages of fast running speed and simple model structure. One of the main advantages of YOLO over other deep learning models is that it is designed for real-time target detection. YOLO can detect targets in an image or video stream in a single pass, making it faster and more efficient than many other models that require multiple passes. YOLO trades off some accuracy for speed compared to other deep learning models. YOLO creatively treats the target recognition task as a regression problem directly and combines Region Of Interest (ROI) and recognition. This allows to detect the location of each disease more precisely, which improves the calculation speed without compromising accuracy. Various types of rice diseases can be observed in paddy fields, such as rice leaf blast, bacterial blight of rice, flax leaf spot and so on. This study aims to improve the accuracy, efficiency, economy and convenience of rice disease diagnosis. The research contents of this article include:

The image dataset was enhanced, and the rice disease recognition model based on YOLOV5s was established to diagnose three different types of rice diseases.

The YOLOV5s model was improved to realize the lightweight of the network, as well as to solve the problem of slow recognition speed caused by large model weight files and calculation amount when the model is deployed in mobile terminals.

## Materials & Methods

### Data acquisition

**Figure 1:**
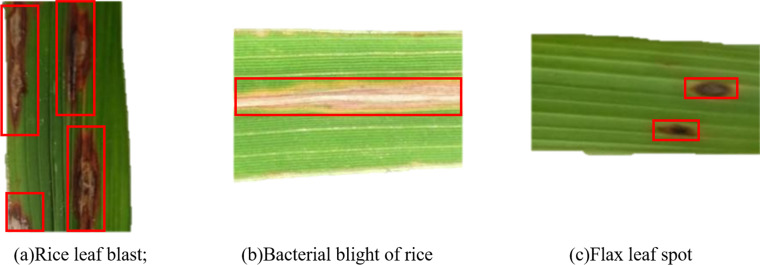
Pictures of rice leaf diseases. (A) Rice leaf blast; (B) bacterial blight of rice; (C) flax leaf spot.

The data set of rice disease pictures used in the experiment was taken by mobile phone, and the pixels were 2400*1080. Each picture has the same size and lighting conditions. The pictures were taken at the Rice Experimental Base in Jinzhai County, Lu’an City, Anhui Province on August 31, 2021. The data set of rice diseases images includes three common diseases, including 1,607 images of rice leaf blast (RLB), 3,174 images of rice bacterial blight (BBR) and 3,235 images of rice flax leaf spot (FLS), with a total of 7,220 images. Figure 1 shows the examples of these three rice leaf diseases. The characteristics of rice leaf blast are that the center of the lesion is grayish white, the edge is brown, and the outer side has a pale-yellow halo. When there are many disease spots, they will form large irregular spots. Bacterial blight of rice is characterized by short strips, most commonly found at the tips and edges of the middle and lower leave. They are small and narrow in shape, yellowish-brown in color and become long strips after expansion. If rice flax leaf spot disease occurs during the germination period, the bud sheath turns brown, and the leaves will die before the rice buds are heading. If it occurs at the seedling stage of rice, the lesions on leaves and sheaths are usually oval-shaped, such as the size of grains and dark brown.

The data set of rice disease pictures are divided into training sets and testing sets, of which about 70% (5,545) are used as training sets and about 30% (2,471) are used as testing sets. Table 2 shows the categories and divisions of the data sets.

**Table 2:** Distribution of data sources and division of training set and validation set.

Categories	Shooting by us	Training set	Validation set	Data enhancement
Rice leaf blast	1,607	1,105	502	4,098 (22.2%)
Bacterial blight of rice	3,174	2,199	975	8,094 (43.86%)
Flax leaf spot	3,235	2,241	994	6,264 (33.94%)
Total	7,220	5,545	2,471	18,456

### Image preprocessing

Image preprocessing and data enhancement. As shown in Fig. 1, the short side of the image is scaled to 640 pixels and the long side is scaled proportionally before reading the image. The image is then subjected to random affine transformations, which can be randomly translated, rotated, scaled, deformed, and cut. Meanwhile, Mosaic data enhancement and image flipping are applied. Finally, the processed image was randomly cropped to a square area of 640*640 pixels as the actual training image. This preprocessing process is beneficial for expanding the data set, reducing the over-fitting of the model and the computation of the model.

**Figure 2:**
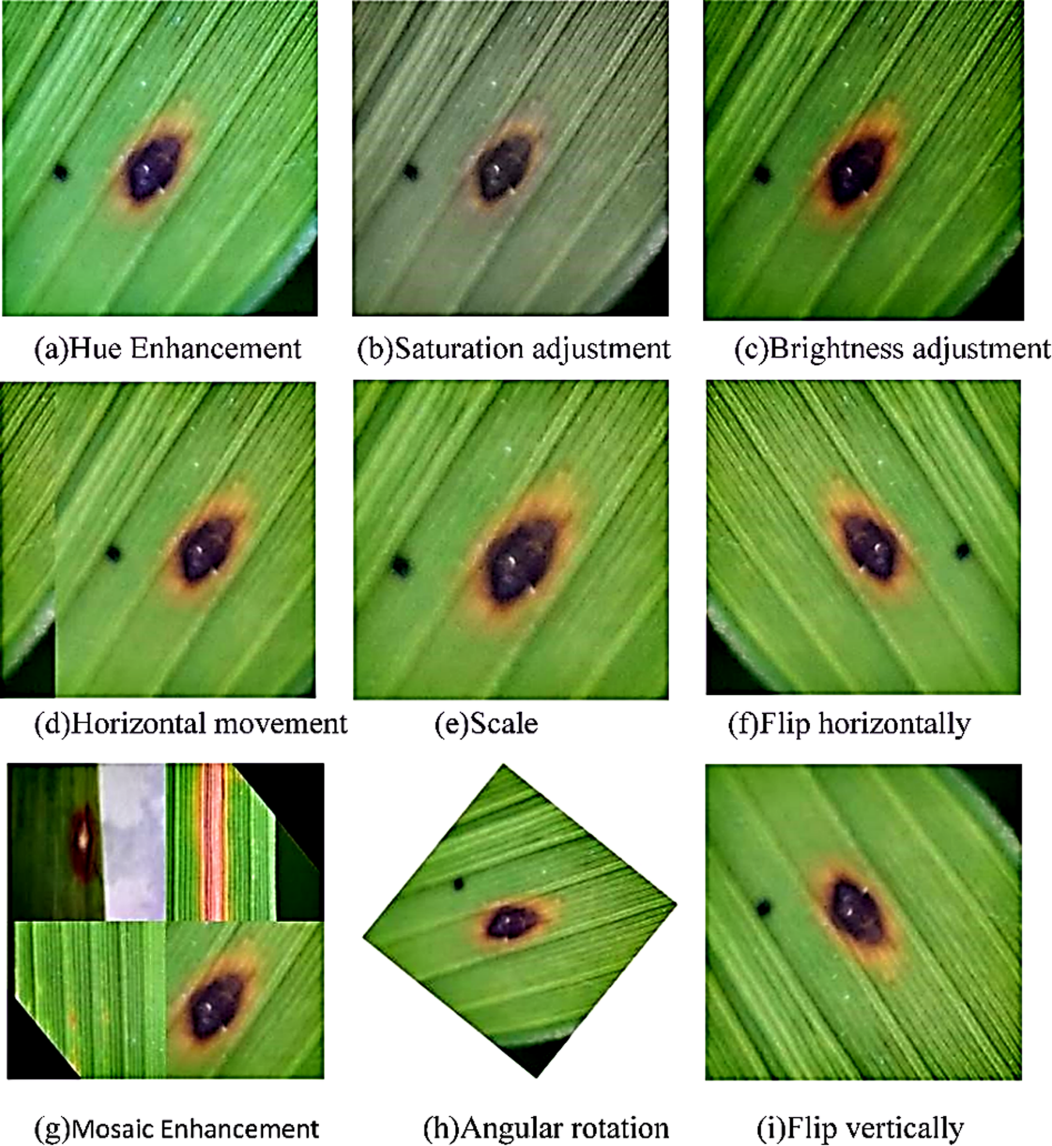
Data enhancement result display. (A) Hue enhancement, (B) saturation adjustment; (C) brightness adjustment; (D) horizontal movement; (E) scale; (F) flip horizontally; (G) Mosaic enhancement; (H) angular rotation; (I) flip vertically.

Figure 2 shows the data set of rice diseases after data enhancement. Since the data enhancement hyperparameter is probability enhancement, it does not mean that the following pictures will appear in the training set. Finally, the number of pictures expanded from 7,220 to 18,456.

### Model design and development

### YOLOV5s network module

YOLOV5 is a one-stage target recognition algorithm proposed by Glenn Jocher in 2020 (Thuan, 2021). There are four versions of YOLOV5, which are YOLOV5s, YOLOV5m, YOLOV5l and YOLOV5x (Wu, Wang & Liu, 2021). Among them, the model size of YOLOV5s network is about one-tenth of the YOLOV4 network, which has faster recognition and location speed, and the accuracy is no lower than YOLOV4 network. As this research has high requirements on the real-time and lightweight performance of the model, we plan to improve the YOLOV5s network architecture on the basis of comprehensive consideration of the accuracy, efficiency, and size of the model.

**Figure 3:**
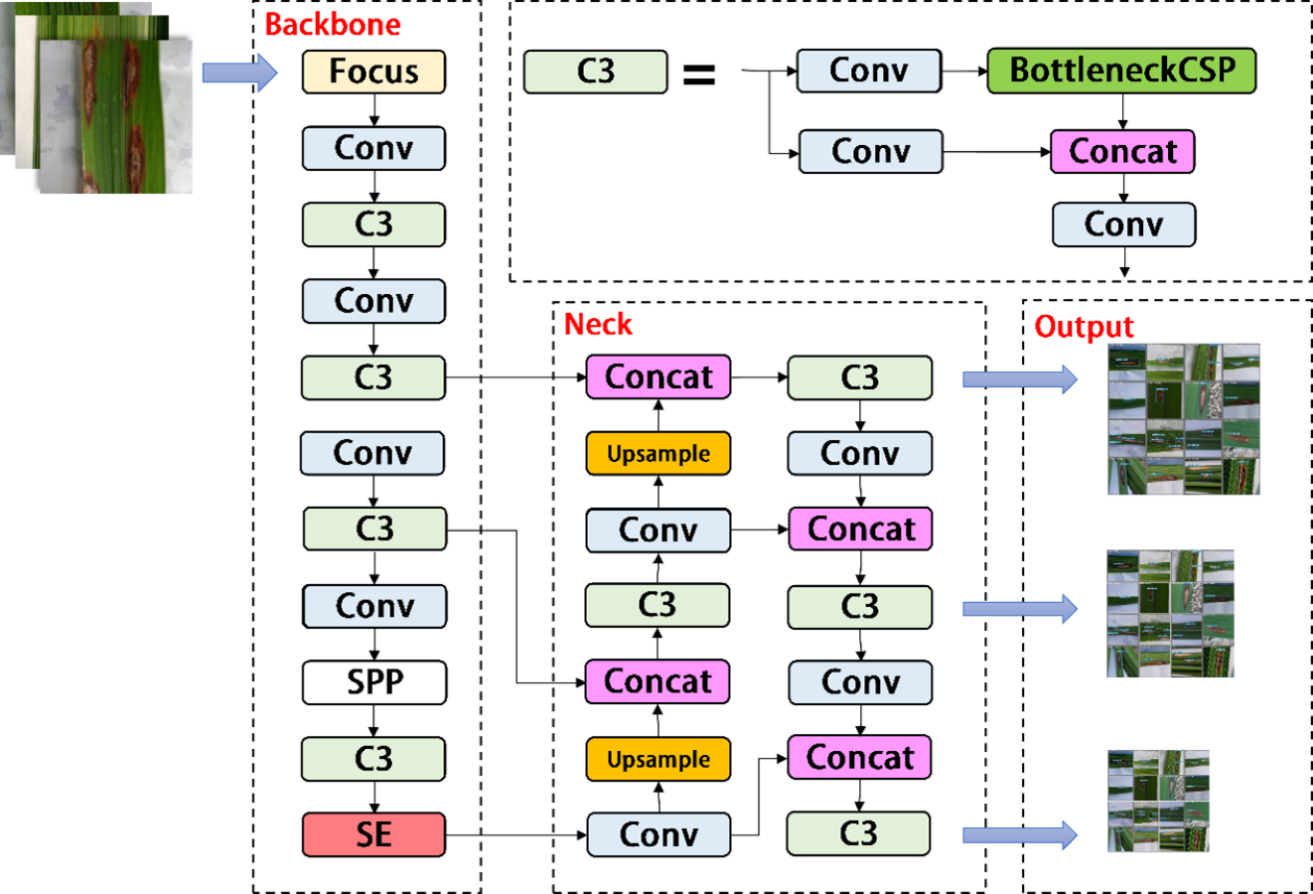
Architecture of the original YOLOv5s network.

As shown in Fig. 3, the YOLOV5s network consists of three main components, including the backbone, neck, and head. After inputting an image, the backbones are aggregated with different image granularities to form image features. Then, the neck sutures the image features and transmits them to the prediction layer, and the head predicts the image features to generate boundary boxes and prediction categories. The YOLOV5s network uses Generalized Intersection over Union (GIOU) as the network loss function, as shown in (1).

(1) \begin{eqnarray*}GIOU=IOU- \frac{{|}C-(A\cup B){|}}{{|}C{|}} \end{eqnarray*}

A, B ⊆ S ⊆ R ^n^, C ⊆ S ⊆ R ^n^, the Intersection over union (IOU) means the ratio of the intersection of A and B to their union. When the input network predicts image features, we use the loss function GIOU and the non-maximum suppression algorithm to filter the optimal target frame.

### YOLOV5-V2 network module design

**Figure 4:**
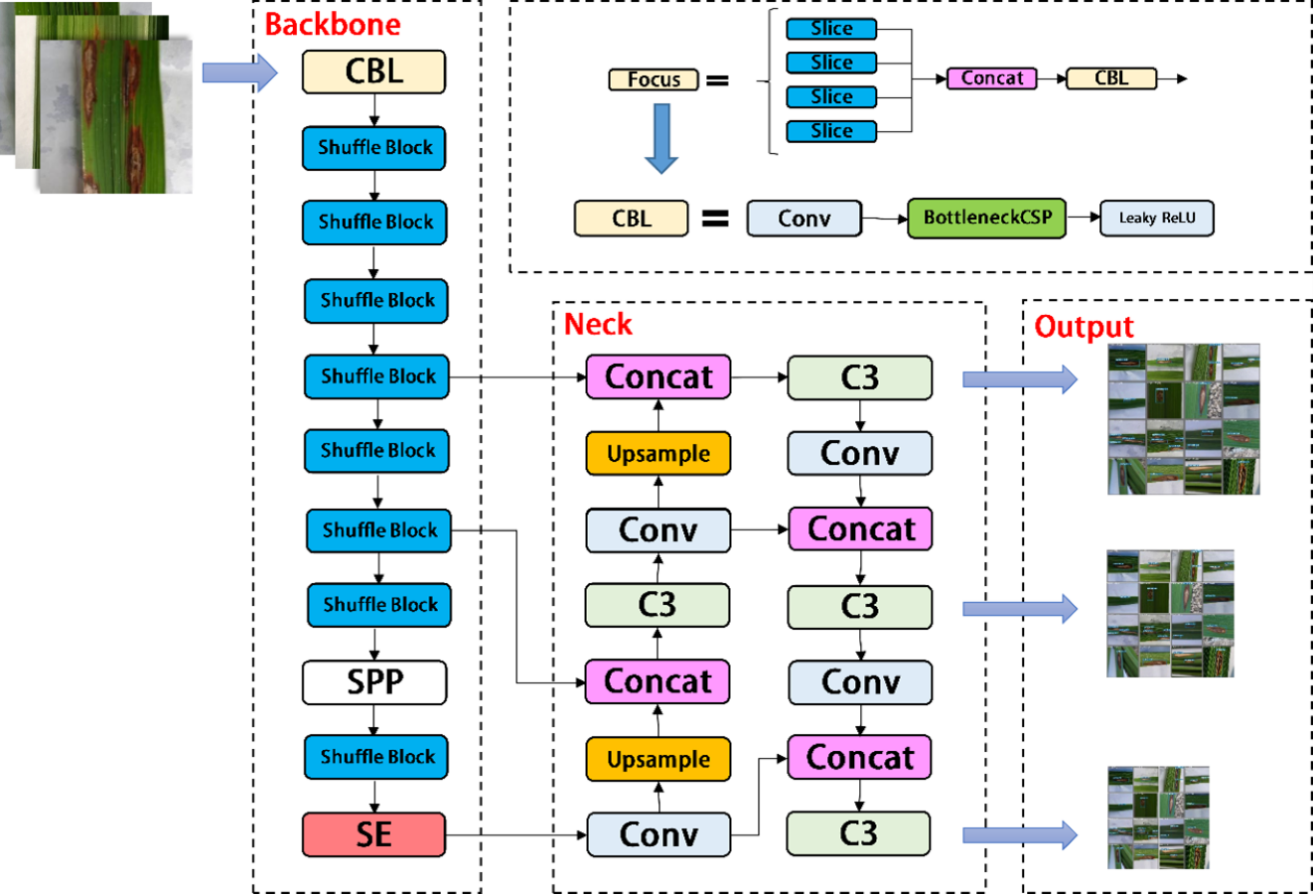
Architecture of YOLOV5-V2 network.

In the backbone of YOLOV5, frequent slice operations in the Focus layer will seriously occupy the cache and increase the burden of computing. In the YOLOV5-V2 network structure designed in this article, the Focus layer of YOLOV5 is deleted to avoid multiple slice operations. C3 Layer, a modified version of BottleneckCSP, is simpler, faster, and lighter, and yields better results at nearly similar loss. However, frequent use of C3 Layer with a high number of channels will take up more cache space and slow down the running speed. In order to avoid this problem, we use the Shuffle block module to replace C3 module in the backbone of YOLOV5-V2, which reduces the number of network parameters and helps to obtain long-distance information of spatial range. At the same time, the goal of reducing the cache and improving the model operation speed is achieved by reducing the channel size. The improved design of the lightweight identification network architecture for rice leaf diseases is shown in Fig. 4.

### YOLOV5-P Network Module Design

**Figure 5:**
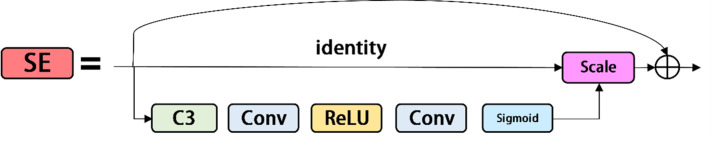
Se modules.

The squeeze-and-excitation network is a network model proposed by Hu, Shen & Sun (2018), which focuses on the relationship between channels. The aim is to learn each image feature according to the loss function, increase the weight of valid image features, and reduce the weight of invalid image features, so as to train the network model to produce the best result. The SE modules is shown in Fig. 5. The SE module is a calculation block, which can be built on the transformation between the input feature vector X and the output feature map U. The transformation relationship is shown in Formula (2):

(2) \begin{eqnarray*}{u}_{c}={v}_{c}\ast X=\sum _{s=1}^{{C}^{{^{\prime}}}}{v}_{c}^{s}\ast {x}^{s}\end{eqnarray*}

where ∗ represents convolution, v _c_ =[ ${v}_{c}^{1},{v}_{c}^{2},\ldots ,{v}_{c}^{{c}^{{^{\prime}}}}$], X =[*x*^1^, *x*^2^, …, *x*^*C*′^] and u _c_ ⊆R ^H×W^. The 2D spatial kernel, represented by ${v}_{c}^{s}$, denotes a single channel of v _c_ that acts on the corresponding channel of X. By adding the SE module to the last layer of the backbone, the image features of powdery mildew and anthracnose are merged in a weighted manner to improve network performance at a small cost.

**Figure 6:**
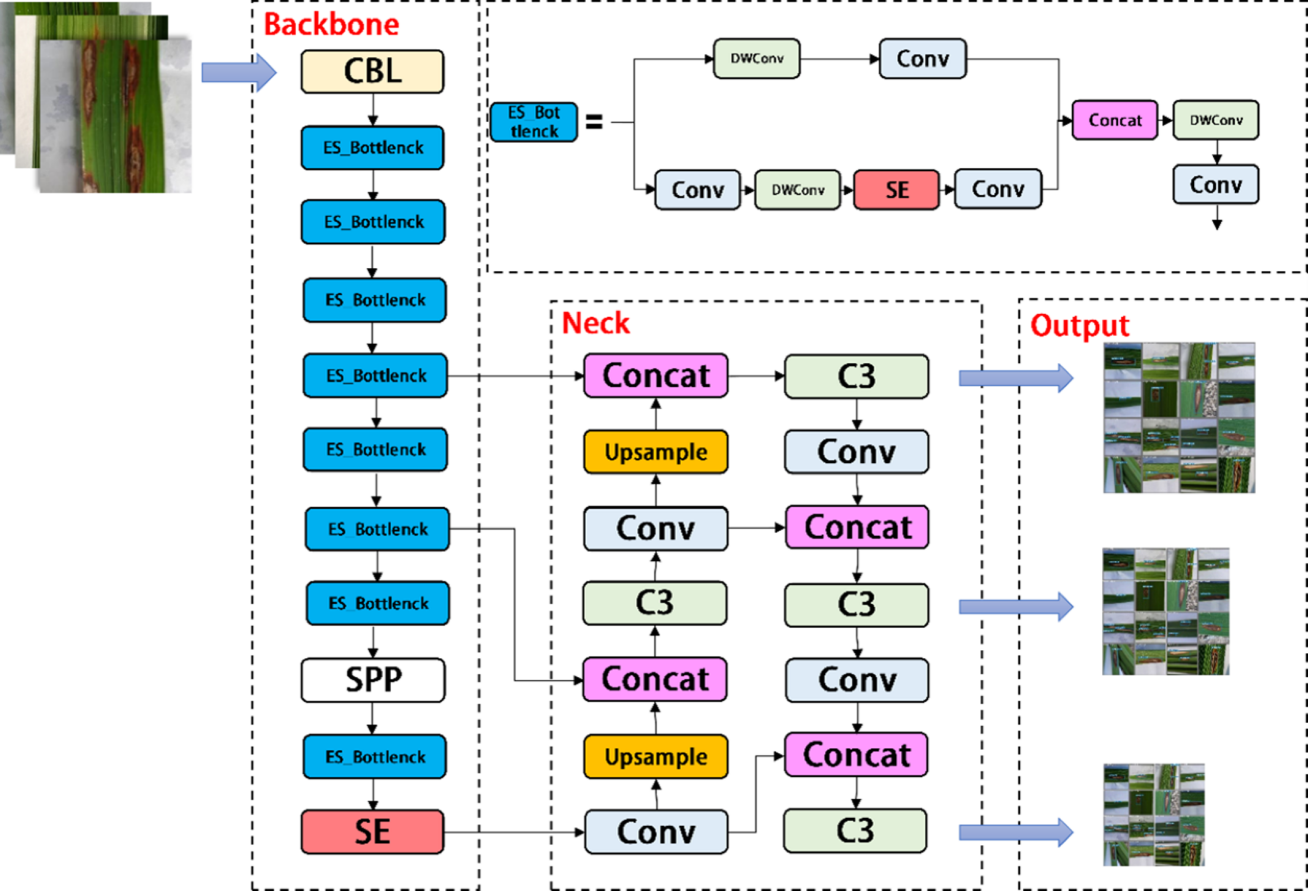
Architecture of YOLOV5-P network.

YOLOV5-P is another network model designed by us, which migrates PP Picodet network on the basis of YOLOV5s. ESNet is the backbone network of PP Picodet, which differs from Shufflenetv2 in that ESNet removes shuffle channel and adds a 3x3 depthwise separable conv. In addition, it adds SE module to one of the branches. We named it ES_Bottlenck. The YOLOV5-P network removes the Focus layer and replaces C3 with ES_Bottlenck, which also aims to reduce the number of parameters and increase the speed of computation. The SE module was added to the last layer of the backbone, allowing it to merge the image features of diseases in a weighted manner, thereby improving the network performance at a small cost. Combined with the ES_Bottlenck and the SE modules, the whole improved YOLOV5s network model framework is constructed, as shown in Fig. 6.

### Evaluation of the Models

The model evaluation indexes used in this article, mainly include precision, Recall, mAP, weights, FPS and Loss.

Precision means that the correctly predicted sample is divided by all the samples, and the formula is as follows:

(3) \begin{eqnarray*}P=TP/(TP+FP)\end{eqnarray*}

where P represents the proportion of positive examples in the examples classified as positive. True Positive (TP) means positive cases are correctly predicted as positive cases; False Positive (FP) means other types of diseases are predicted as positive cases.

Recall is a measure of coverage. To measure how many positive examples are predicted to be positive, the formula is as follows:

(4) \begin{eqnarray*}recall=TP/(TP+FN)\end{eqnarray*}

where False Negative (FN) means negative disease is predicted to be another disease, and TP+FN is equal to the real number of targets in target recognition.

mAP is the average precision of all categories divided by the sum of all categories, *i.e.,* the average of the average precision of all classes in the dataset. mAP is defined as follows:

(5) \begin{eqnarray*}\text{mAP}=\text{the sum of the average precision of all categories/all categories}.\end{eqnarray*}

Weights refers to the memory size of files obtained after training, and the memory size will directly affect the recognition rate of mobile deployment.

Loss is used to evaluate the difference between the predicted value and the actual value of the model.

FPS is to detect several frames of images per second, that is, the reciprocal of the time to detect one frame of images. The formula is as follows:

(6) \begin{eqnarray*}\text{FPS}=1/\text{detection time}.\end{eqnarray*}

### Experimental equipment

The program used in this study was developed in Python 3.8 and ran on a desktop computer with Ubuntu 18.04 as the operating system. The hardware included an Intel(R) Xeon(R) CPU E5-2678 v3 processor, 11GB of memory, and a GeForce RTX 2080Ti graphics card. The Pytorch framework and YOLOV5 environment were built in the Anaconda3 environment, with a CUDA version of 11.0. The specific configurations are provided in Table 3.

**Table 3:** Test environment setting.

Parameter	Configuration
Operating system	Ubuntu 18.04
Deep learning framework	Pytorch 1.7.1
Programming language	Python 3.8
GPU accelerated environment	CUDA 11.0
GPU	GeForce RTX 2080Ti 11G
CPU	Intel(R) Xeon(R) CPU E5-2678 v3 @ 2.50 GHz

### Testing process

**Figure 7:**
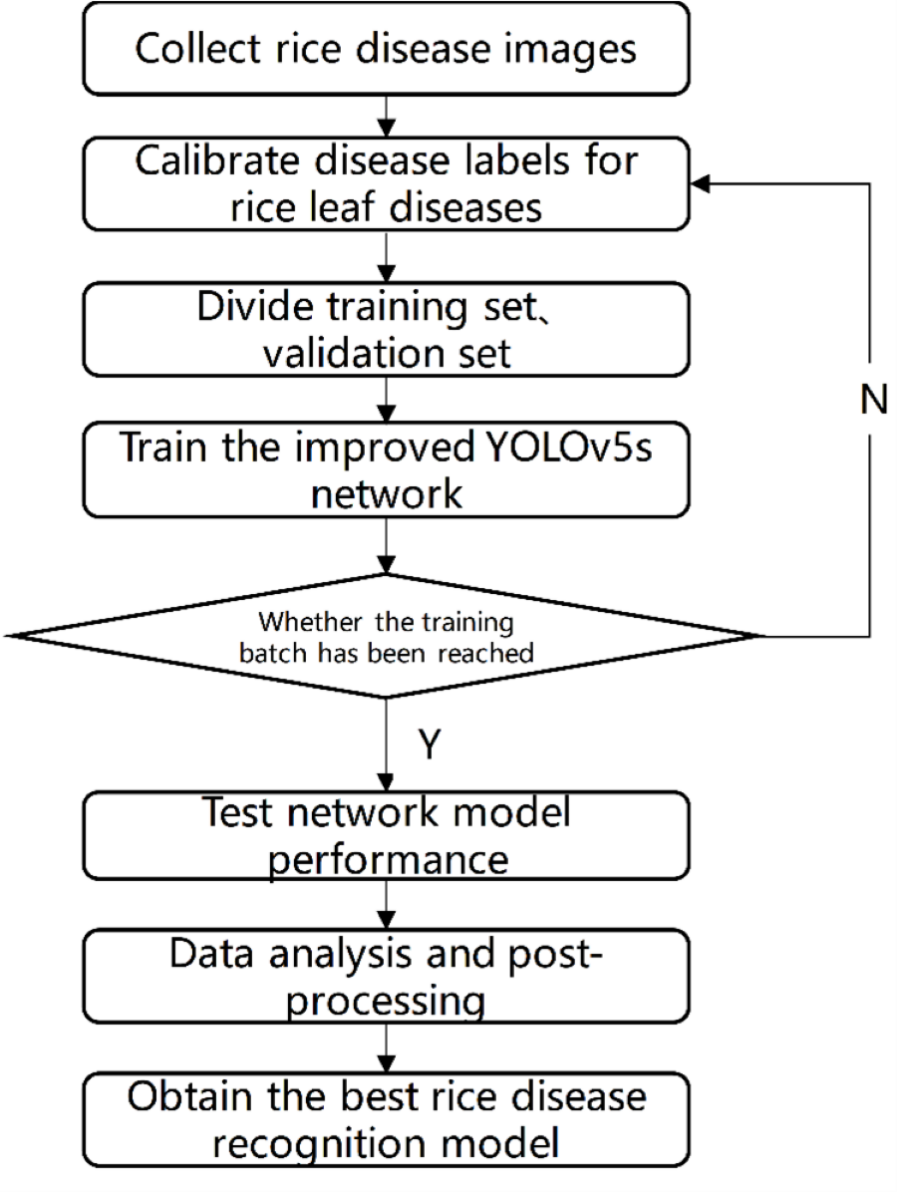
Test flow chart.

The test process is shown in Fig. 7. Firstly, images of rice diseases were obtained, and then the data were divided into three categories according to the characteristics of disease spots. The image was clipped to a uniform size of 640*640, and then label the processed data set with the software named labelImg. Afterwards, the disease image set was divided at a 7:3 ratio into training and validation sets. The training process involved inputting the training set into the improved YOLOV5 network with different structures and optimizing the network model using the Stochastic Gradient Descent algorithm. After completing the 300 batches of training, the optimal network weight was obtained. To evaluate the performance of the network model, the test set was used and compared with the results of the original YOLOV5 and our two improved versions. The network model with the best result was selected as the rice disease recognition model.

The labeled images were input into the YOLOV5 model, and then the super-parameter evolution mechanism of YOLOV5 was used to enhance the rice disease data set. Then the expanded data set was input into YOLOV5s, YOLOv4, YOLOR, YOLOv3 and Faster RCNN for training. During the training process, we found that some models can set batch size to be larger. For example, YOLOV5 and YOLOR set batch size to 32. However, the batch size of YOLOv3, YOLOv4, and Faster RCNN can only be set to 16, 8, and 8, respectively, due to the memory capacity of the graphics card. Finally, by comparing the precision, weight, mAP, recall, loss and other indicators, the model suitable for mobile deployment with high recognition rate was selected, so that the rice disease target recognition task can be accomplished efficiently.

## Results

### Convergence Results of the Network Model

In order to further analyze the recognition performance of various algorithms for rice diseases, this article compares the experiments of YOLOV5-V2, YOLOV5, YOLOV5-P on 200 test set images.

**Figure 8:**
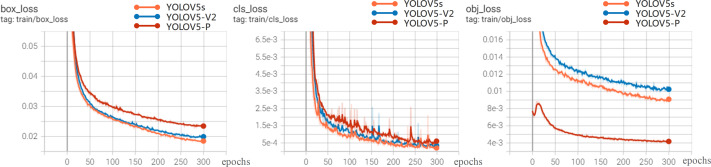
Loss experimental results.

Figure 8 shows the loss curve. The overall trend of the loss value is that it drops rapidly in the first 300 epochs and basically tends to be stable at 200 epochs, indicating that the model is well designed and without over-fitting. Therefore, the model after 300 training sessions is determined as the rice disease identification model. It can be seen from the loss curve that the performance of YOLOV5-V2 and YOLOV5s models is better than that of YOLOV5-P in the loss function curve.

### Results and analysis of improvement of YOLOV5s network architecture

**Figure 9:**
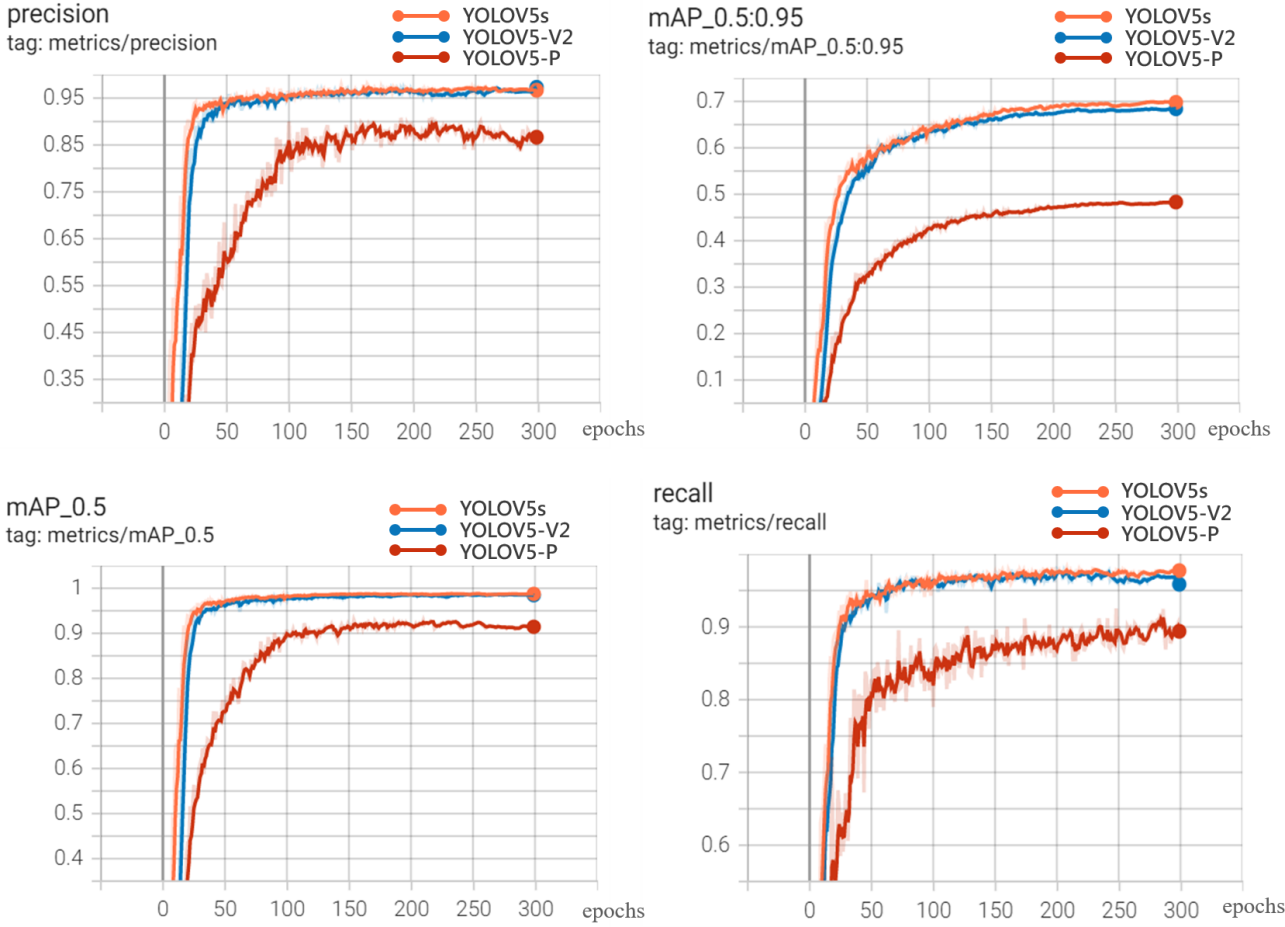
Experimental results.

The visualization results of recognition performance are shown in Fig. 9, with mAP and average recognition speed as the evaluation indexes of the model.

The comparison results of evaluation indexes between the two improved models and YOLOV5s model are shown in Table 4.

**Table 4:** Improved statistical results of YOLOV5 model experiments.

Model	Size (Pixels)	mAP-0.5	mAP-0.5:0.95	Precision	Recall	FPS	Average detection speed (s/img)	Weights (MB)
YOLOV5s	640*640	**98.83%**	**70.01%**	**97.96%**	**98.19%**	22.5	0.044	13.8
YOLOV5-V2	640*640	98.65%	68.53%	97.64%	97.92%	**69.0**	**0.014**	3.2
YOLOV5-p	640*640	92.93%	48.45%	90.92%	97.92%	40.0	0.025	**2.2**

**Notes.**

Best results are shown in bold.

It can be seen from Fig. 9 and Table 4 that YOLOV5s is 0.32%, 0.27%, 0.18% and 1.48% higher than YOLOV5-V2 in precision, recall, mAP-0.5 and mAP-0.5:0.95, respectively. However, the weight file of YOLOV5-V2 model is 3.2MB, which is 76.81% less than that of YOLOV5s. Its average detection speed is 0.014 s per image, and the prediction time of a single image is about 1/3 of that of YOLOV5s. This model can meet the requirements of real-time disease recognition and is conducive to the deployment of mobile terminals. The performance of YOLOV5-P is not satisfactory. Although the weight of the model is only 2.2MB, and the prediction time of a single image is about 1/2 less than that of YOLOV5s. The performance of precision, Recall and mAP is not satisfactory. Therefore, comprehensively, YOLOV5-V2 network not only ensures the accuracy of identification but also effectively realizes the lightweight characteristics of the network.

**Figure 10:**
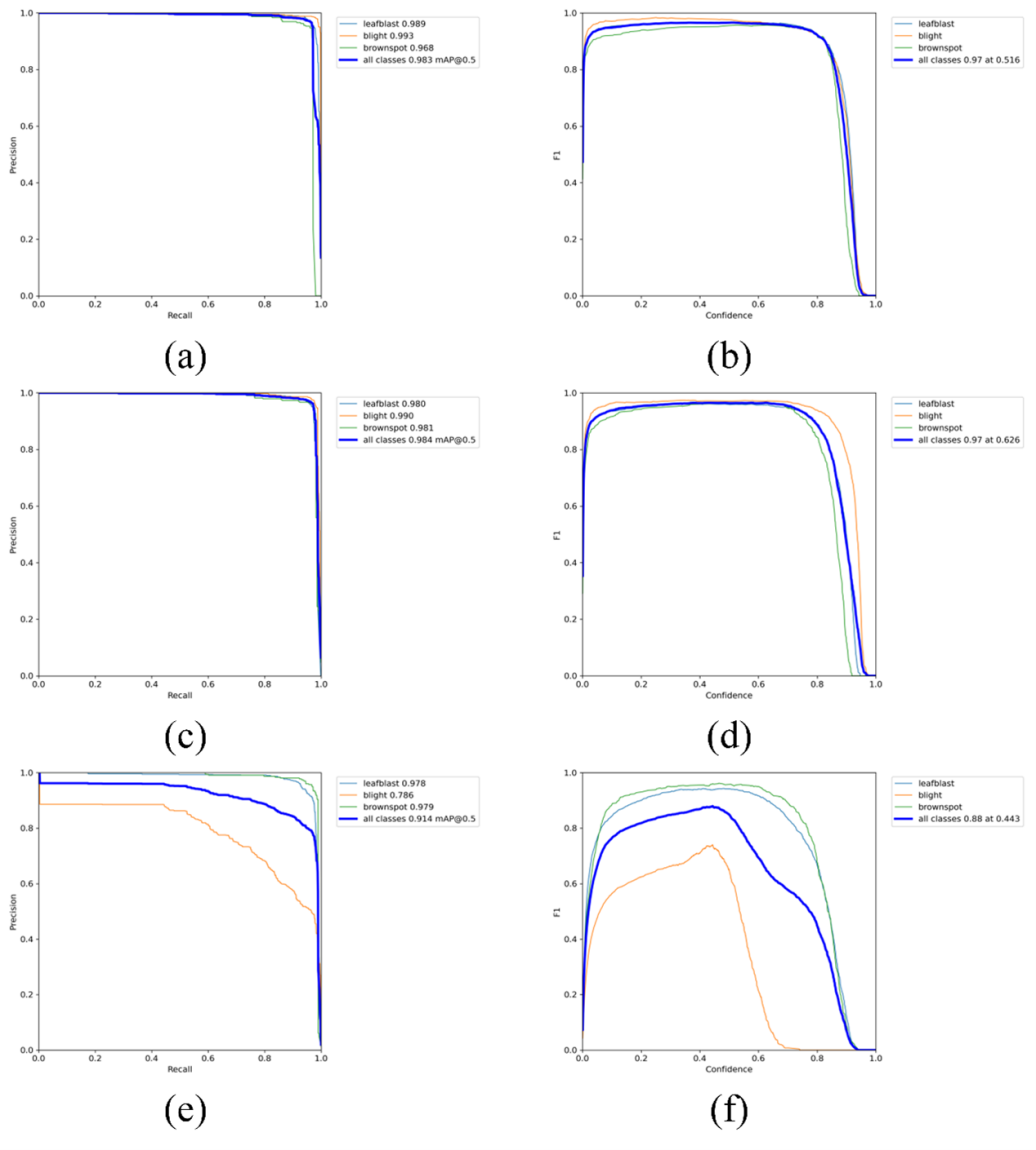
(A–F) PR curve graph and F1 curve graph.

Figure 10 is the PR_curve graph and F1_curve graph of YOLOV5s, YOLOV5-V2 and YOLOV5-P. F1_curve refers to the relationship between F1 score and confidence degree. The optimal prediction threshold is determined by adjusting the confidence degree threshold and combining with the requirements of target recognition task. As can be seen from Fig. 10, YOLOV5-V2 and YOLOV5s have very good identification effects on the three rice diseases, while YOLOV5-P has good performance in the curve of rice leaf blight and flax leaf spot and a poor performance in the curve of bacterial blight.

**Figure 11:**
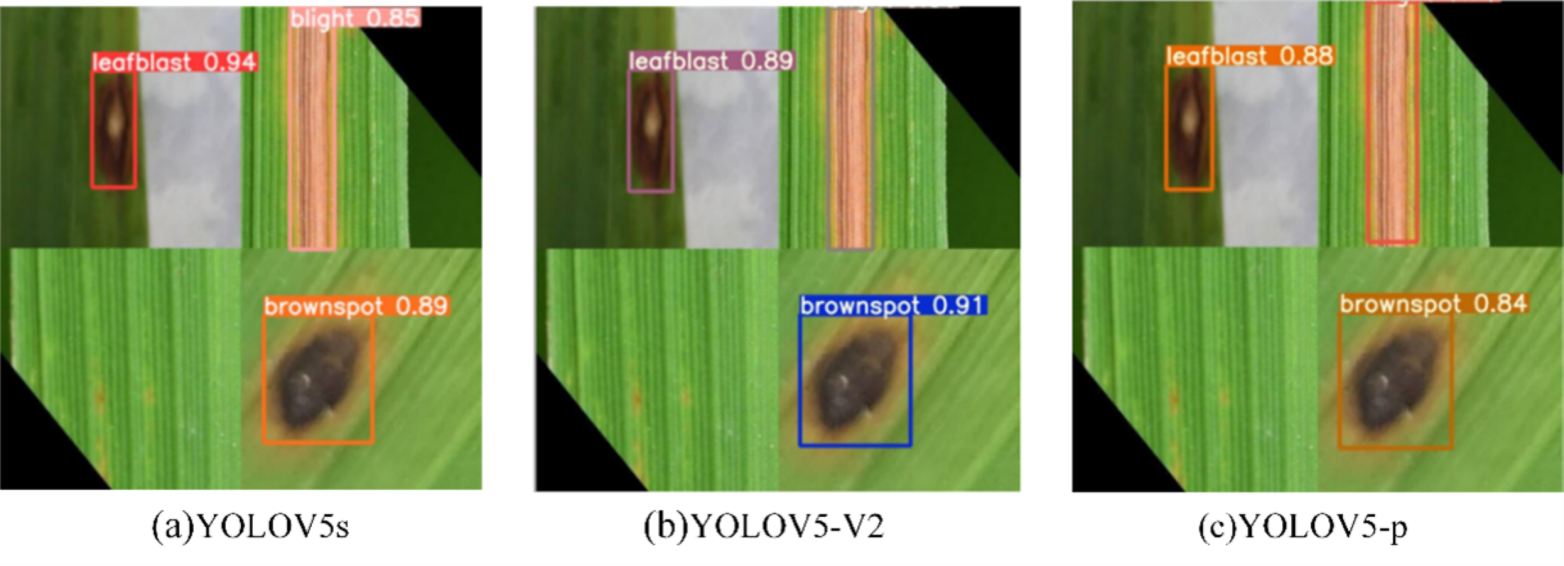
(A–C) Comparison of prediction of three experimental rice diseases.

The recognition results of the three model test images are shown in Fig. 11. Consistent with the above analysis, there is no significant difference between YOLOV5s and YOLOV5-V2 in recognition effect, while YOLOV5-P has poor recognition effect and the lowest recognition accuracy.

## Discussion

### Results compared with other models

In order to compare the performance of different networks in rice disease diagnosis, a variety of network structures were selected here, including Faster RCNN, YOLOV3, YOLOV4, YOLOR, YOLOV5s, and two improved models. The map_0.5, Precision, recall, model size and other results obtained in the experiment are shown in Table 5.

**Table 5:** Performance comparison of seven network models.

Model	mAP	Precision	Recall	Weights (MB)
SVM	78.58%	73.10%	72.40%	/
Faster RCNN	92.38%	92.16%	92.30%	184
YOLOV3	98.98%	98.12%	98.02%	118
YOLOV4	98.88%	93.14%	98.78%	141
YOLOV5s	98.83%	97.96%	98.19%	13.8
YOLOV5-V2	98.65%	97.64%	97.92%	3.2
YOLOV5-P	92.93%	90.92%	97.92%	2.2

As can be seen from Table 5, YOLOV3 has the highest mAP and recognition accuracy, reaching 98.98% and 98.12%. MAP and accuracy of YOLOV5-V2 are 98.65% and 97.64% respectively, which are only 0.33% and 0.48% lower than the maximum value. However, the weights of YOLOV5-V2 is 3.2M, which is greatly reduced compared with 118M of YOLOV3. To sum up, the improved model YOLOV5-V2 in this article has the best comprehensive performance in recognition performance of three types of diseases. On the premise of ensuring high accuracy, it has fewer model parameters and faster calculation, which can meet the requirements of real-time performance and is conducive to the deployment of mobile terminals.

### Technical challenges

There are several technical challenges in deep learning for rice disease identification that should be addressed, including:

One of the main challenges in developing deep learning models for rice disease identification is the limited availability of labeled training data. Deep learning models require large amounts of labeled data to learn the features that distinguish between different rice diseases, but collecting and annotating such data can be time-consuming and expensive.

Deep learning models are often considered black-box models, which means that it can be difficult to understand how the model arrives at its predictions. This can be a significant challenge for rice disease identification, where it is important to understand which features the model is using to make its predictions. Techniques such as visualizing feature maps and attention mechanisms can help to increase model interpretability.

## Conclusions

In this article, a data set containing images of three kinds of rice diseases was established, and a lightweight real-time detection method of rice diseases based on improved YOLOV5s was proposed. In the improved network architecture C3 module is replaced by shuffle block module, named YOLOV5-V2 in order to realize the lightweight of the network. In addition, the C3 module was replaced with ES_Bottlenck and the SE module was inserted in the backbone named YOLOV5-P. At last, the comparative experiments were carried out with Faster RCNN, YOLOV3, YOLOV4, YOLOV5s, YOLOV5-V2 and YOLOV5-P, respectively. Visual analysis confirmed satisfactory learning ability of YOLOV5-V2 model for rice disease characteristics. The accuracy of the model is 97.64% through the test of independent sources image, indicating that the model has sufficient generalization ability. In addition, the weight of the model file is small, which can be implemented in the application of rice disease diagnosis.

##  Supplemental Information

10.7717/peerj-cs.1384/supp-1 Supplemental Information 1 YOLOV5s model

10.7717/peerj-cs.1384/supp-2 Supplemental Information 2 YOLOV5s network module A lightweight version of the YOLOv5 object detection algorithm designed to be efficient and suitable for resource-constrained environments. The ’runs’ file contains the experimental results of the model on the rice disease dataset.

10.7717/peerj-cs.1384/supp-3 Supplemental Information 3 YOLOv5 object detection algorithm A lightweight version designed to be efficient and suitable for resource-constrained environments. The ’runs’ file contains the experimental results of the model on the rice disease dataset.

10.7717/peerj-cs.1384/supp-4 Supplemental Information 4 YOLOV5-P model This model migrates PP Picodet network on the basis of YOLOV5s.

## Additional Information and Declarations

### Data Availability

The following information was supplied regarding data availability: The data is available at figshare: (2022): dataset. figshare. Figure. https://doi.org/10.6084/m9.figshare.21514515.v1. The code is available in the Supplementary Files.
